# Colistin Insusceptibility in Carbapenem-Resistant Enterobacteriaceae Isolates From a Tertiary Referral Centre

**DOI:** 10.7759/cureus.71329

**Published:** 2024-10-12

**Authors:** Payal S Hait, Satyajeet K Pawar, Satish R Patil

**Affiliations:** 1 Department of Microbiology, Krishna Institute of Medical Sciences, Krishna Vishwa Vidyapeeth, Satara, IND

**Keywords:** antimicrobial resistance, broth micro-dilution, carbapenem-resistant enterobacteriaceae, colistin resistance, modified carbapenem inactivation method

## Abstract

Background

Antimicrobial resistance has developed significant importance as a worldwide health concern more so in the 21^st^ century. This is more specifically observed among the *Enterobacteriaceae* family, the major group of Gram-negative bacteria. Resistance to carbapenems and colistin antimicrobials from the reserve group, in critical infection treatment poses a substantial therapeutic challenge. This study aims to detect resistance to both carbapenems and colistin in *Enterobacteriaceae* isolates, emphasizing the importance of monitoring.

Materials and methods

A laboratory-based study investigated 82 Carbapenem-Resistant *Enterobacteriaceae* (CRE) isolates from hospitalized patients at Krishna Hospital & Medical Research Centre, Karad, India, for two years (November 2021-November 2023). The Kirby-Bauer disc diffusion method was used to determine the antimicrobial susceptibility following Clinical Laboratory Standards Institute 2022 recommendations. To validate Carbapenem-Resistant *Enterobacteriaceae* isolates, the Modified Carbapenem Inactivation Method (mCIM) was used, and Colistin resistance was ascertained using Broth Microdilution Susceptibility Testing (BMD).

Results

Among 309 *Enterobacteriaceae* isolates, 82 (26.5%) exhibited carbapenem resistance, with 75 confirmed by Modified Carbapenem Inactivation Method (mCIM) testing. For patients in the age group 51-60 years (20%), with men being the most prevalent, the predominance of CRE isolates was from the Intensive Care Unit (44%), mostly from urine samples (34.6%). The most dominant CRE was *Klebsiella pneumoniae* (62.7%), followed by *Escherichia coli* (32%). Colistin resistance was found in 14 (18.7%) of CRE isolates, with the highest minimum inhibitory concentration (MIC) at 4 µg/mL (n=10) and 16 µg/mL (n=4). Colistin-resistant isolates (n=10) were sensitive to amikacin (71.4%) and resistant to many antibiotics.

Conclusion

The extreme rise of colistin-resistant cases among CRE in healthcare settings is a solemn alarm. Among the most effective treatments, aminoglycosides amikacin and netilmicin show the greatest sensitivity against these colistin-resistant CRE infections. The findings stress the difficulties in treating such infections and the dire need for solid antimicrobial stewardship and novel therapeutic strategies.

## Introduction

Over the past two decades, antimicrobial resistance has become a major worldwide health concern, particularly among Gram-negative bacteria like *Enterobacteriaceae*. Bacteria have developed a variety of resistance mechanisms, rendering several classes of antibiotics ineffective. Therefore, this phenomenon has led to the rise of infections resistant to numerous antibiotic classes [1].

Antimicrobial resistance is a substantial contributor among Gram-negative bacteria, particularly those belonging to the *Enterobacteriaceae* family. This family characterizes the most heterogeneous and largest group of Gram-negative bacilli which is frequently recovered from clinical samples. The *Enterobacteriaceae *family is ubiquitous, and widely distributed in soil, water, and plants, and the natural microbiota of both human and animal digestive tracts. Clinically, they are responsible for sepsis cases, urinary tract infections, and a majority of intestinal, hospital, and community-acquired infections, including meningitis, pneumonia, and surgical wound infections [1].

In treating Gram-negative bacterial infections, carbapenems, a class of β-lactam antimicrobial agents, have become a vital option, especially in intensive care settings due to their extended range of activity. The rise of Carbapenem-Resistant *Enterobacteriaceae* (CRE), which are *Enterobacteriaceae* resistant to one or more of the carbapenems, i.e., ertapenem, meropenem, imipenem, or doripenem asserts a critical challenge. The development of resistance mechanisms in these bacteria is due to carbapenemase production, overexpression of efflux pumps, loss of porins in the bacterial cell membrane, and reduced binding of carbapenems to penicillin-binding proteins [1].

 A cationic polypeptide antibiotic, colistin, is one of the oldest antibiotics from the polymyxin group, and it is used as a last-resort treatment for CRE infections. Its antimicrobial mechanism involves disrupting the bacterial cell membrane by interacting with colistin amino groups and lipid A subunits of lipopolysaccharides, leading to cell death. Despite its effectiveness, resistance mechanisms to colistin have been increasingly reported, complicating treatment options. Therefore, a critical threat is concurrent resistance to carbapenems and colistin, making monitoring and controlling the spread of these resistant bacteria essential [2].

So, it's veritably important to stop the escalation of those bacteria having resistance against both CRE and colistin. The purpose of this study was to detect resistance against both carbapenems and colistin in *Enterobacteriaceae* isolates.

## Materials and methods

This laboratory-based investigation was carried out from November 2021 to November 2023 at the Department of Microbiology, Krishna Institute of Medical Sciences and Krishna Hospital & Medical Research Centre, Karad. The Ethics Committee of Krishna Institute of Medical Sciences approved this study (Protocol number 072/2021-2022). The study analyzed 82 CRE isolates. Data were gathered from the admitted patients of Krishna Hospital with suspected infections, utilizing a structured proforma to document patient demographics and clinical information. The analysis includes all clinical specimens received throughout the study period, including pus, endotracheal secretions, sputum, urine, cerebrospinal fluid, blood, body fluids, and additional samples such as catheter tips and knee aspirates.

Isolation and antimicrobial susceptibility testing

Specimens were cultivated on both Blood agar and MacConkey agar and incubated overnight at 37°C. Standard methodologies were employed to identify isolated bacterial colonies [3]. The Kirby-Bauer disc diffusion method was performed for antimicrobial susceptibility testing (AST) on Mueller Hinton agar (MHA) plates in compliance with Clinical and Laboratory Standards Institute (CLSI) guidelines 2022 [4]. The bacterial inoculum suspension was standardized to 0.5 turbidity using the McFarland standard. After inoculation, antibiotic discs were positioned on the agar surface using sterile forceps within a quarter of an hour. The zone of inhibition was determined using a standardized scale after incubation of plates for 24 hours at 37°C, and according to the CLSI, results were interpreted [4].

Phenotypic detection of carbapenem-resistant *Enterobacteriaceae* by modified carbapenem inactivation method (mCIM)

Under CLSI guidelines, an imipenem disc was used to perform the mCIM test. A suspension of bacterial colonies from an overnight blood agar bacterial culture was prepared in 2 ml of tryptone soya broth (TSB), and then a 10 µg imipenem disc was added to each broth. The suspension was incubated at 37°C for four hours. Following this, a suspension of 0.5 McFarland turbidity of *Escherichia coli* ATCC 25922 was prepared and spread evenly onto Mueller Hinton Agar (MHA). The imipenem disc from the TSB suspension was carefully blotted to remove excess broth and then placed on the MHA plate, inoculated with an indicator strain of *Escherichia coli*. The plates were incubated for 18 to 24 hours at 37°C [5]. Positive (*Klebsiella pneumoniae* ATCC BAA-1705) and negative (*Escherichia coli *ATCC 25922) controls were used to ensure result accuracy, with interpretation conducted per CLSI [4].

Broth micro-dilution susceptibility testing (BMD)

Cation-adjusted MHA (CAMHB) was used for cultivating Gram-negative isolates, with pH adjusted to 7.2-7.4 for conducting Minimum Inhibitory Concentration (MIC) tests. Antimicrobial susceptibility testing adhered to CLSI guidelines, using standard drug powder calibrated to 19,000 units/mg potency. Drug potency was determined before testing, and stocks were prepared and stored in cryo vials. These stocks were used to prepare working solutions at 4X concentration. Susceptibility testing at various dilutions was performed using microtiter plates. Growth in wells containing antibiotics was compared to control wells to determine susceptibility. The results were evaluated according to CLSI 2022 criteria as follows: ≤2 µg/ml is considered sensitive, and >2 µg/ml is considered resistant [6]. Positive (*Escherichia coli* NCTC 13846) and negative (*Escherichia coli *ATCC 25922) controls were used to ensure result accuracy, with interpretation conducted per CLSI guidelines [4].

## Results

**Figure 1:**
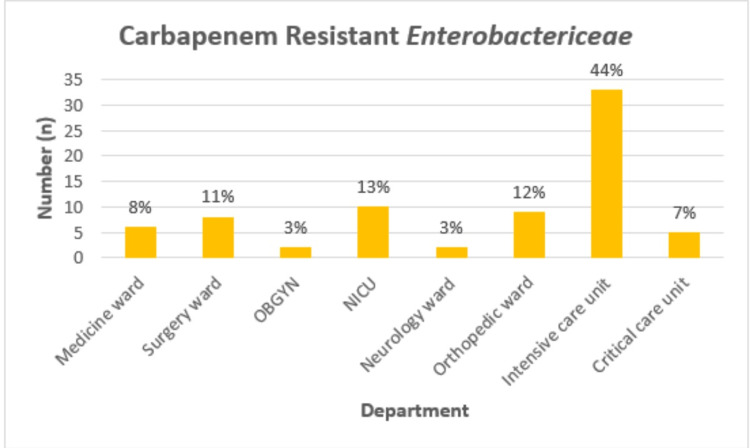
Carbapenem-resistant Enterobacteriaceae distribution across various departments OBGYN: Obstetrics and gynecology; NICU: Neonatal intensive care unit.

In the study, 309 isolates of *Enterobacteriaceae* were tested during one year, with 82 (26.53%) identified as carbapenem resistant using the Kirby Bauer disc diffusion method. Of these, 75 isolates (91.4%) were confirmed through the mCIM test, and colistin resistance was assessed using the BMD test on these confirmed isolates. These 75 CRE isolates were derived from hospitalized patients across various wards and clinical specimens. The study showed maximum isolates were reported from the age group 51-60, which was 20% containing nine males and six females (n=15) presented that male is the predominant gender. The study showed that the maximum number of CRE isolates were from the Intensive Care Unit (44%) (Figure 1).

**Figure 2:**
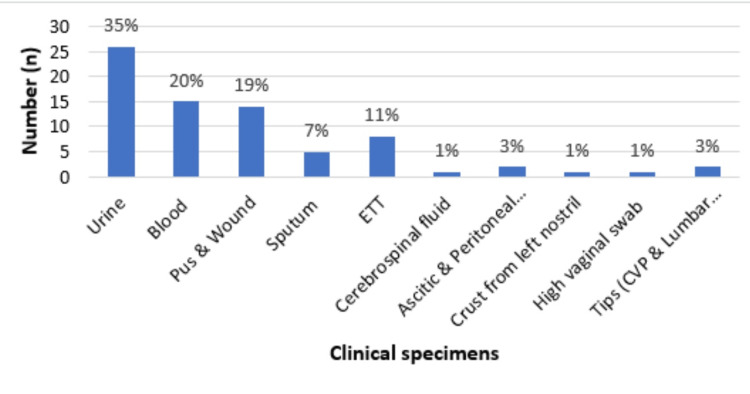
Carbapenem-resistant Enterobacteriaceae distribution in different clinical specimens ETT: Endotracheal tube; CVP: Central venous pressure.

Figure 2 shows that the maximum number of isolates were obtained from clinical specimen urine (34.6%), followed by blood (20%), pus, and wound (19%).

Out of 75 isolates, *Klebsiella pneumoniae* (62.7%) was the maximum CRE isolate, followed by *Escherichia coli* (32%), *Klebsiella oxytoca *(4%), and *Enterobacter cloacae complex *(1.4%) (Table 1).

**Table 1:** Distribution of microorganisms among carbapenem-resistant Enterobacteriaceae isolates

Bacteria	Number (n)	Percentage (%)
Escherichia coli	24	32
Klebsiella pneumoniae	47	62.7
Klebsiella oxytoca	03	4
Enterobacter cloacae complex	01	1.4
Total	75	100

Out of 75 confirmed CRE isolates, 14 (18.7%) showed colistin resistance by BMD test, and 61 (81.3%) were sensitive among hospitalized patients.

A MIC value of 4 µg/mL was shown by 10 (13.3%) colistin-resistant CREisolates, while a MIC value of 16 µg/mL was shown by four (5.4%) colistin-resistant CRE isolates. Among sensitive isolates, 0.5 µg/mL MIC was found for a maximum of 24 (32%) isolates (Table 2).

**Table 2:** Colistin MIC value for CRE isolates by broth microdilution MIC: Minimum inhibitory concentration; CRE: Carbapenem-resistant Enterobacteriaceae.

No. of CRE isolates	MIC Value									
	0.03	0.06	0.12	0.25	0.5	1	2	4	8	16
75	0	0	3 (4%)	19 (25.4%)	24 (32%)	10 (13.3%)	5 (6.7%)	10 (13.3%)	0	4 (5.4%)

**Figure 3:**
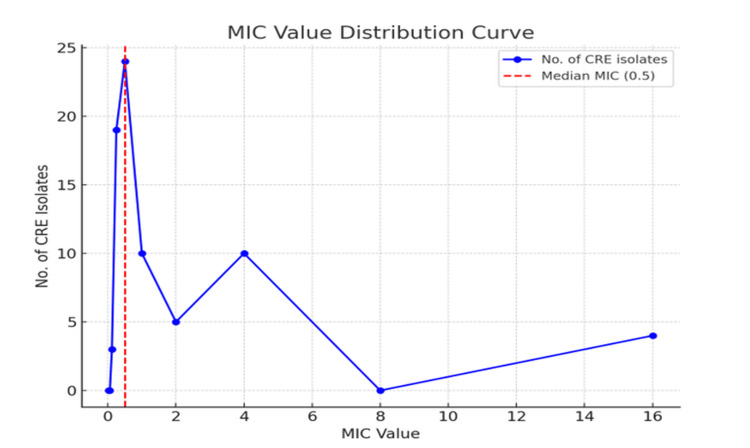
Median distribution curve of colistin MIC value for CRE isolates by broth microdilution MIC: Minimum inhibitory concentration; CRE: Carbapenem-resistant Enterobacteriaceae; No.: number.

Fifty-three (70.7%) of the CRE isolates had a MIC value of ≥ 0.5 µg/mL (Median Minimum Inhibitory Concentration value), as shown by the median distribution curve (Figure 3).

Out of 14 colistin-resistant CRE isolates, *Klebsiella pneumoniae* (71.4%) was the maximum CRE isolate, followed by *Escherichia coli* (21.4%) and *Klebsiella oxytoca* (7.2%) (Table 3).

**Table 3:** Distribution of colistin-resistant organisms among isolates CRE: Carbapenem-resistant Enterobacteriaceae.

Bacteria	Number (n)	Percentage (%)
Escherichia coli	3	21.4
Klebsiella pneumoniae	10	71.4
Klebsiella oxytoca	1	7.2
Total	14	100

**Figure 4:**
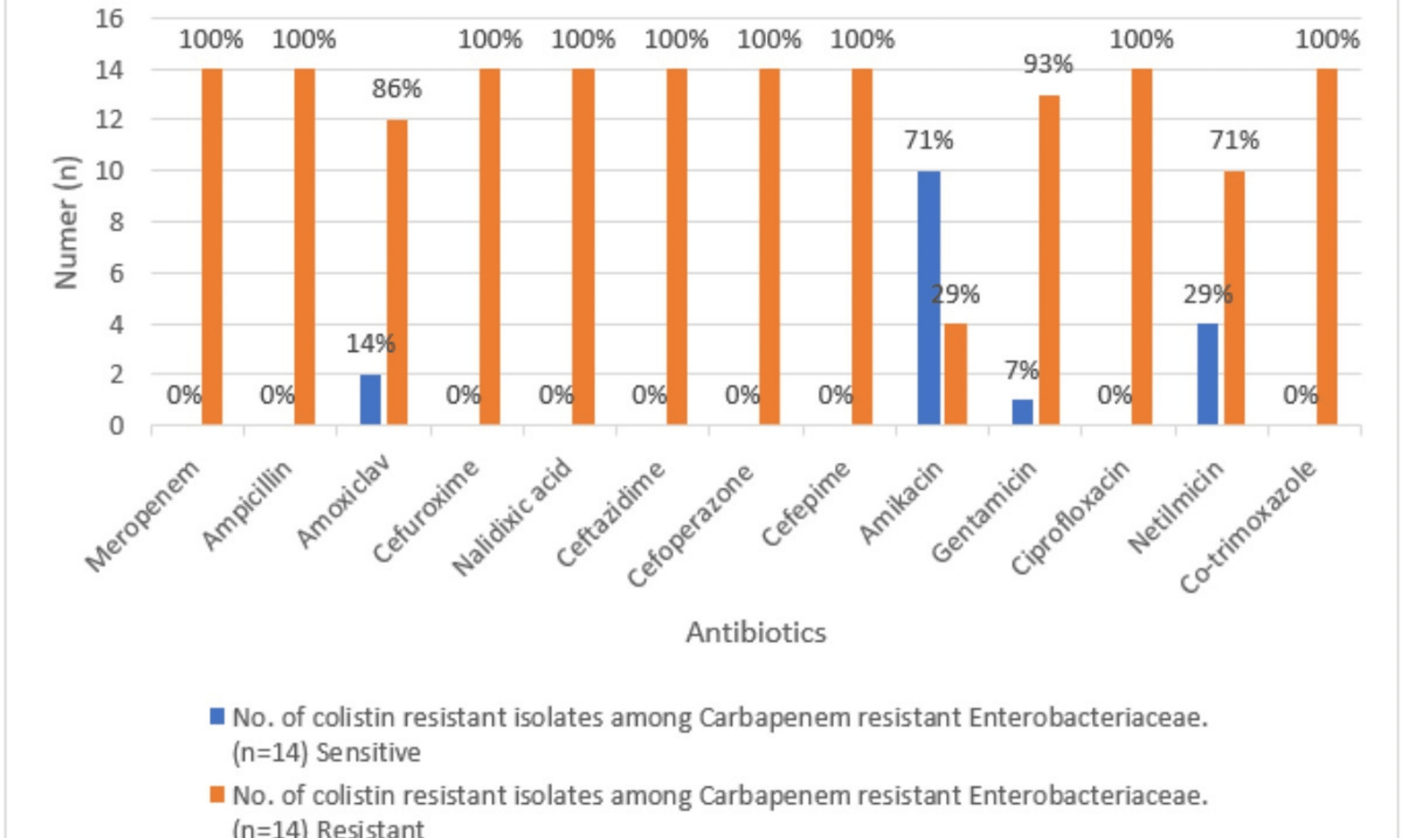
Antibiogram of colistin resistant isolates among CRE isolates amoxiclav: amoxicillin-clavulanate; No.: number

Colistin-resistant CREisolates showed the highest sensitivity to amikacin (n=10) which was 71.4% and maximum resistance to ampicillin, cefuroxime, nalidixic acid, ceftazidime, cefoperazone, cefepime, ciprofloxacin, and co-trimoxazole (n=14) which was 100% (Figure 4).

## Discussion

One of the major concerns of today's medicine deals with antibiotic resistance development [7]. Microorganisms have manifested the astonishing ability to adapt and develop complex mechanisms, allowing protection of their genetic material and continue passing through generations, assuring the preservation of their species [8]. The progression of antibiotic-resistant Gram-negative bacteria stands as excellent evidence of the potential these organisms have to pick up, retain, and express novel genetic material that confers antibiotic resistance. This rise underscores the pressing requirement for infection prevention measures that limit their spread [7].

Of all the isolates, the highest records were regained in the ICU, followed by the neonatal intensive care unit (NICU) and orthopedic surgery, critical care medicine surgery, and medicine units. The fewest numbers of isolates were recovered from the obstetrics gynecology (OBGYN) and neurology wards. The distribution of isolates of *Escherichia coli* and *Klebsiella pneumoniae* from clinical samples according to the treatment units, as reported by Nagaraj et al., demonstrated that the ICU, general surgery, and general medicine had comparatively higher concentrations of carbapenem-resistant strains [9]. These two results corroborate the notion that the contributions of the ICU, in general, and critical care units, in particular, are major reservoirs for bacterial isolates. This evidences these as key nodes for the spread of infections within the interior environment of hospitals, exemplifying targeted control efforts strategies.

The present study reports a maximum number of its isolates were from urine samples 26 (34.6%), followed by blood samples 15 (20%), pus and wound samples 14 (18.7%), endotracheal tube samples eight (10.6%), etc. Kar et al. documented that 109 (54.5%) isolates were from urine specimens, followed by respiratory specimens 42 (21%), and from pus 24 (12%) [10]. In the study by Giri et al., the major source was urine with 44 (29.33%); blood, 30 (20%); pus, 17 (11.33%); sputum, nine (6%); ascitic fluid, five (3.33%); wound swab, four (2.67%); and pleural fluid, two (1.33%) [11]. This reflects the dominance of urine specimens among the sources of the isolates. 

In this study, 75 CRE* *were isolated and recognized as follows: *Klebsiella pneumoniae* (47); *Escherichia coli *(24); *Klebsiella oxytoca* (3); and *Enterobacter cloacae complex *(1). However, according to the study by Pragasam et al., most of the isolates were carbapenemase-producing *Escherichia coli* (n=34) and carbapenemase-producing *Klebsiella pneumoniae* (n=55) from the family *Enterobacteriaceae* [12]. Contrary to the study by Kar et al., CRE species included *Escherichia coli*, which was the main species of CRE, at 56.5%, followed by *Klebsiella pneumoniae*, accounting for 39.5%, while *Enterobacter spp*. contributed 4% [10]. Thus, in this current study, *Klebsiella pneumoniae* was a major CREspecies, contrary to the reports of Pragasam et al. and Kar et al., which showed *Escherichia coli *as the most prevalent species. This portrays the wide variation in the distribution of CRE species between these studies. The *Enterobacteriaceae* family organisms are among the dominant organisms of the gut flora. For such organisms, carbapenemase production is mostly because of the horizontal transfer of plasmid-borne genes. Such dissemination could happen via fecal-oral route or contact, whether it is community or hospital-acquired infections. Samples of urine from hospitalized patients, particularly those who had undergone catheterization, demonstrated a higher prevalence of CRE. Poor hand cleanliness habits in surgical patients serve as one of the contributing factors for such high prevalence. Longer duration of hospital stays and exposure to invasive procedures are regarded as significant risk variables for CRE infection among ICU patients [1,13-15].

The present study shows that CRE isolates were resistant to colistin 14 (18.7%) and sensitive to 61 (81.3%) in the hospital-admitted patients. However, in a study reported by Kar et al., colistin resistance was detected in 27 out of 200 CRE isolates, 13% by the reference BMD method. The majority were contributed by *Klebsiella pneumoniae* (81%), followed by *Escherichia coli* (11%) [10]. A comparatively higher prevalence was discovered in this study compared to Kar et al. As per the research reported by Qadi et al., results indicated 59% of isolates were susceptible, and 41% were resistant [14].

A MIC value of 4 µg/mL was shown by 10 (13.3%) colistin-resistant CRE isolates, while a MIC value of 16 µg/mL was shown by four (5.4%) colistin-resistant CREisolates. Among sensitive isolates, 0.5 µg/mL MIC was found for a maximum of 24 (32 %) isolates. Kar et al. showed that the majority of the MIC values from the BMD test were > 8 µg/mL, accounting for 7.5%, followed by 8 and 4 µg/mL, at 3% each [10]. Colistin resistance was most prominent at the MIC value of 16 µg/mL, with a rate of 5.3%, while a considerable resistance rate at a MIC value of 4 µg/mL was 13.3%. There is a great need for a unified way of testing antimicrobial resistance to produce consistent and accurate results from different studies. The higher resistance to the highest MIC values indicates an increasing resistance to colistin in this hospital setting. It is not surprising that the animal sector has contributed to the emergence and worldwide spreading of colistin resistance, most notably in countries that have long used colistin as an enhancer of development in animal husbandry [16].

Among these 14 colistin-resistant CRE isolates, maximally sensitive to amikacin was 71.4% (n=10), followed by amoxicillin-clavulanate (amoxiclav) that was 14% (n=2) and all resistant to ampicillin, cefuroxime, nalidixic acid, ceftazidime, cefoperazone, cefepime, ciprofloxacin, and co-trimoxazole. Intracellular penetration of aminoglycosides is a complex process involving, although not limited to, passive diffusion via porin channels, transport mediated by carriers whose functioning depends on the electron transport chain; these are, in turn, impacted by elements such as oxygen availability and pH levels [17]. These 14% of isolates sensitive to amoxiclav can be attributed to two primary mechanisms of carbapenem resistance. First, the production of β-lactamases, such as extended-spectrum β-lactamases (ESBLs) or derepressed cephalosporinases, with minimal carbapenemase activity, combined with reduced permeability due to porin loss, may not effectively degrade amoxicillin-clavulanate (amoxiclav). Second, some isolates produce carbapenem-hydrolyzing β-lactamases, but clavulanate can inhibit certain β-lactamases, particularly those with weaker carbapenemase activity. Furthermore, efflux mechanisms may not completely affect amoxicillin-clavulanate action but instead strengthen resistance to other antibiotics [18]. Colistin resistance in bacteria is mediated by several molecular mechanisms. Resistance acquired to colistin is related to a modification in lipopolysaccharide (LPS) based on alteration of outer membrane porins and decreasing of lipopolysaccharide (LPS) negative charge, as well as up-regulation of efflux pumps and overproduction of capsule polysaccharide. The resistance is mediated by mcr genes such as mcr-1 transposable genetic elements [2,19].

Limitations

Genotypic detection of colistin resistance was not studied in the present study. The data collected is primarily demographic and does not include detailed patient follow-up information in the present study.

## Conclusions

The spike in overall colistin-resistant cases among CRE within a healthcare environment is a serious concern. To balance the high risk to health in nosocomial settings, extreme precautions should be put into operation, and other alternatives to therapy must be considered. Among the most effective treatments, aminoglycosides, amikacin and netilmicin, show the greatest sensitivity against these colistin-resistant CRE infections. The findings stress the difficulties in treating CRE and colistin-resistant infections as a dire need for solid antimicrobial stewardship and novel therapeutic strategies. Understanding the mechanisms of resistance and efficiencies of antibiotics, such as amikacin, is of high importance in devising efficacious treatment approaches against resistance to multiple drugs.
